# Food hygiene practice and associated factors among food handlers working in food establishments in sub-Saharan Africa: a systematic review and meta-analysis

**DOI:** 10.1017/S146342362500009X

**Published:** 2025-02-21

**Authors:** Yibeltal Assefa Atalay, Natnael Atnafu Gebeyehu, Kelemu Abebe Gelaw

**Affiliations:** 1 School of Public Health, College of Health Science and Medicine, Wolaita Sodo University, Wolaita Sodo, Ethiopia; 2 School of Midwifery, College of Health Science and Medicine, Wolaita Sodo University, Wolaita Sodo, Ethiopia

**Keywords:** Factors, food handlers, food hygiene, good practices, sub-Saharan Africa, systematic review

## Abstract

**Introduction:**

Food hygiene practices are crucial to avoid foodborne illness and improve human well-being. Millions of people get sick, and many of them pass away due to eating unhealthy food. Foodborne diseases are still a public health problem in developing countries.

**Objective:**

This study aimed to determine the prevalence and factors associated with food hygiene practices among food handlers in sub-Saharan Africa.

**Methods:**

An extensive search was conducted using various databases including PubMed, Science Direct, African Journal Online, and Google Scholar. The search results were then extracted using Microsoft Excel. The data analysis was conducted using STATA version 14. Publication bias was checked by funnel plot, and more objectively through Begg and Egger regression test, with P < 0.05 considered to indicate potential publication bias. A random effect model was used to calculate the pooled prevalence of hygienic food handling practices. Sub-group analysis was done by country and study site.

**Results:**

To estimate the pooled prevalence of food hygiene practices in sub-Saharan Africa, 42 reviewed studies and 12,367 study participants were included. The pooled prevalence of food hygiene practices among food handlers in sub-Saharan Africa was found to be 50.68% (95% CI: 45.35, 56.02) in this study. Factors associated with food hygiene practices included lack of food safety training (OR = 2.14 95% CI: 0.68, 6.76), negative attitude (OR: 2.36, 95% CI: 1.36, 4.09), and lack of regular medical checkups (OR: 2.66, 95% CI: 1.52, 4.65) among food handlers.

**Conclusion:**

This research found that only half of sub-Saharan Africa’s food handlers had good food hygiene practices. Lack of food safety training, a lack of regular medical checkups, and unfavorable attitudes toward food hygiene practices were factors contributing to food hygiene practices. Thus, the authors recommended that food workers receive food safety training about food hygiene and safety procedures.

## Introduction

Food hygiene is an essential matter of public health for protecting or preventing diseases caused by unsafe food due to lack of good quality from production, processing to consumption (Ethiopian Ministry of Health, 2019). Food safety (or food hygiene) is used as a scientific method/discipline that describes the handling, preparation, and storage of food in a manner that prevents foodborne disease (Food Safety Definition and Why Is Food Safety Important, 2018).

According to the World Health Organization (WHO), one in 10 people worldwide suffers from foodborne illnesses, endangering both developed and underdeveloped countries. Consumption of contaminated food poses a significant threat to billions of people worldwide (Fung et al., 2018). Each year, an estimated 600 million individuals experience illness as a result of consuming food contaminated with harmful agents, leading to a staggering 420,000 fatalities attributed to the disease (Zanin et al., 2017).

Foodborne diseases have a significant global impact and affect people of all ages, especially children under five years of age. They are more common in developing countries due to a lack of sanitation, a lack of drinking water, contaminated and improper food storage equipment, and a lack of food safety education (Ucar et al., 2016; Stratev et al., 2017; Lamuka, 2014).

Most foodborne illnesses are caused by bacterial, viral, and parasitic infections. *Salmonella*, *campylobacter*, *enterohaemorrhagic Escherichia coli,* and *listeria* are the most common bacteria causing foodborne infections. Other foodborne diseases caused by intestinal parasites such as *Entamoeba histolytic*, *Giardia lamblia, Taenia species*, *Ascaris lumbricoide,* and *Trichuris trichiura* are associated with unsanitary food handling (Kendall, 2022; Belhu et al., 2020).

Institutional and community food service is an important sector of the food industry. Food consumed in such facilities is considered to be a major cause of foodborne disease outbreaks (Parry-Hanson Kunadu et al., 2016). Foodborne illnesses in facilities with large numbers of people pose a public health concern because outbreaks in these locations can affect large numbers of consumers at the same time. In developing countries, the lack of ensuring proper hygienic food handling practices in these areas is a major concern (Abdul-Mutalib et al., 2012).

Food handlers are expected to have excellent hygiene practices to reduce cross-contamination and protect consumers from foodborne diseases (Nnebue et al., 2014). Poor personal hygiene frequently contributes to foodborne illness which indicates that food handlers’ knowledge and handling practices need to be improved (Akabanda et al., 2017). Pooled data studies on the conditions of food and drink establishments have been scanty in sub-Saharan Africa.

Foodborne infections affect the socioeconomic development of these countries. Foodborne bacterial diseases are common in sub-Saharan Africa. Ensuring food hygiene practices contributes to a high level of food safety, the most important aspect of food quality. To protect consumer health, food safety and hygiene are vital (Heman). For this reason, both the European Union and the WHO recommend that community measures such as food safety, food hygiene, and water security be re-evaluated in light of scientific evidence, which is crucial for the prevention of foodborne infections (European Commission, 2018).

Based on our search databases, there is no systematic review and meta-analysis on hygienic food handling practices in sub-Saharan Africa. For this reason, there is a limitation in easy access to compiled documents on hygienic food handling practices and the factors involved. The lack of a pooled study examining the prevalence and factors related to food hygiene practices among food handlers in food businesses represents a significant gap. This review can provide well-organized data that form the start of available research on food handling practices in sub-Saharan Africa.

The objective of this systematic review and meta-analysis was to identify the pooled prevalence of food hygiene practices and associated factors among food handlers working in food establishments in sub-Saharan Africa. What was the status of food hygiene practices at food handlers? And what factors were associated with food hygiene practices among food handlers in sub-Saharan Africa? The results of this study could help governmental and non-governmental organizations to develop and implement effective strategies to improve food hygiene and safety for food handlers.

## Methods

### The study protocol and registration

The purpose of this systematic review and meta-analysis is to determine the pooled prevalence of food hygiene practices and its factors among food handlers in sub-Saharan Africa. To ensure the accuracy and completeness of the study, the Preferred Reporting Items for Systematic Reviews and Meta-Analyses (PRISMA) 2009 checklist was used (Liberati et al., 2009). The review protocol has been submitted to the International Prospective Register for systematic reviews to ensure transparency and accountability.

### Searching strategy

A comprehensive search of databases was undertaken using PubMed, Science Direct, African Journal Online, and Google Scholar to find potentially relevant articles focusing on food hygiene practices and related factors among food handlers in sub-Saharan Africa. In addition to the database search, the cited literature listed in the reference of the articles was also manually searched, and the relevant additional articles were identified and included. The search strategy used the Boolean operators ‘AND’ and ‘OR’ to refine the search results. Keywords used in the search included ‘Food’, ‘food handling Practices’, ‘hand hygiene’, ‘food hygiene’, ‘associated factors’, and ‘sub-Saharan Africa’. These search terms were selected based on the PECCO – principles selected to ensure retrieval of relevant articles from the above databases. All searches were limited to papers written in English, and the last search in all databases was performed on 22 November 2023.

### Population, exposure, context, condition, and outcomes (PECCO) guidelines

P = Population: The food handlers. E = Exposure: The level of exposure plays a crucial role in influencing the adherence to food hygiene practices by food handlers in sub-Saharan Africa. These factors include food safety training, level of education, medical checkups, and food handler attitudes. C = Context: sub-Saharan Africa. C = Condition: hand hygiene practices.

O = Outcome measurement: The main objective of the research was to assess the prevalence of food hygiene practices. Furthermore, the study sought to investigate the factors that impact safety practices among food processors. This goal was accomplished through the analysis of data from primary studies using odds ratio and binary outcomes.

### Inclusion and exclusion criteria

This study included studies that met specific criteria. These criteria included having a population of food handlers, focusing on the prevalence of food hygienic practice and its associated factors. The studies were conducted exclusively in sub-Saharan Africa and were published in English. However, certain primary studies were excluded for various reasons. These reasons included a lack of information on the prevalence of food hygiene practice, unavailability of the full text, low-quality score, inability to access the full text after multiple attempts to contact the corresponding author, and exclusion of narrative reviews, editorials, correspondence, abstracts, or methodological studies.

### Data extraction

Using a pretested data extraction format, two researchers (YAA and KAG) extracted all the required data. The first author or research group name, year of publication, study country, study setting, study design, sample size, and status of hand hygiene practice were all extracted. The reviewers independently collected data on factors associated with hand hygiene practices. For the second outcome (factors related to food hygiene practice), the data were extracted in a 2-by-2 table format and the odds ratio for each factor was calculated based on the findings of the original studies.

### Operational definitions

**Food hygiene practice:** food handlers who scored less than the mean value of the score of the practice questions were considered as having ‘poor food hygiene practices’ and those who scored mean and above the mean value of the practice questions were considered as having ‘good food hygiene practice’ (Abdi et al., 2020).

**Food establishment:** facilities that provide large groups of consumers with food and drink services such as breakfast, lunch, dinner, or cocktails. These institutions include hotels, cafes and restaurants, cafeterias, and butcher shops (Zeleke et al., 2022).

### Data analysis

After extraction of all relevant findings in a Microsoft Excel spreadsheet, the data were exported to STATA software version 14 for analysis. The pooled prevalence of food hygiene practice was calculated using a 95% confidence interval. Publication bias was checked by funnel chart and more objectively by Begg and Eggers regression tests, with P < 0.05 indicating possible publication bias. The presence of heterogeneity between studies was checked using the Cochrane Q statistic. This heterogeneity between studies was quantified using I^2^, in which a value of 0, 25, 50, and 75% represented no, low, medium, and high heterogeneity, respectively. A forest plot was used to visually assess the presence of heterogeneity, which presented at a high-level random effect model was used for analysis to estimate the pooled estimate of food hygiene practice. Sub-group analysis was done by country, study setting, and sampling techniques. A sensitivity analysis was executed to see the effect of a single study on the overall prevalence of the meta-analysis estimate. The findings of the study were presented in the form of text, tables, and figures.

## Results

### Searching process

**Figure 1.**
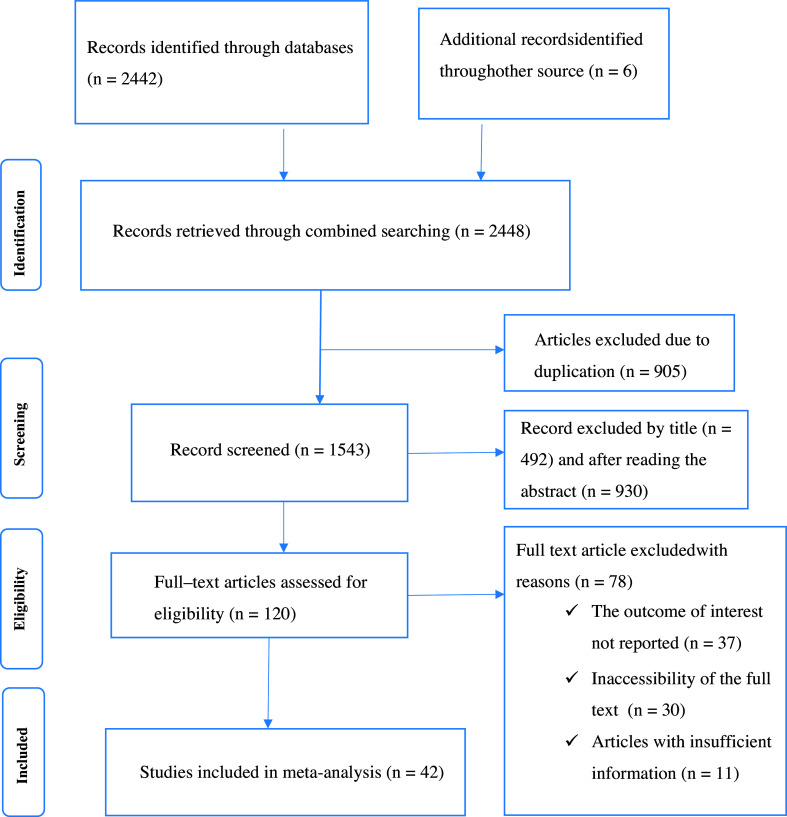
PRISMA flow chart displays the article selection process for food hygiene practice in sub-Saharan Africa.

This systematic review and meta-analysis included published studies conducted on the prevalence and factors associated with hygienic food handling practices among food handlers in sub-Saharan. A total of 2,448 records were retrieved through electronic database searching. After removing duplicated studies, we obtained 1543 studies selected for screening full titles and abstracts. Of these, 1422 studies were excluded due to title and abstracts, and the remaining 120 articles were assessed for full-text articles. After reviewing the full text, 78 articles were then eliminated because they lacked full titles and abstracts and reported findings. Finally, 42 full-text primary articles were selected for quantitative analysis (Fig. 1).

### Characteristics of included studies

This systematic review and meta-analysis included 42 articles with a total sample size of 12,367 food handlers (Blaise, 2014; Mwove et al., 2020; Fanta et al., 2023; Abegaz, 2022; Oladoyinbo et al., 2015; Werkneh et al., 2023; Negassa et al., 2023; Jumanne & Sophia, 2014; Engdaw et al., 2023; Tuglo et al., 2021; Makhunga et al., 2023; Teferi et al., 2021; Marutha & Chelule, 2020; Mbombo-Dweba et al., 2022; Azanaw et al., 2019; Ndoli & Nicholas, 2019; Alemu et al., 2023; Nonga et al., 2014; Tegegne & Phyo, 2017; Akabanda et al., 2017; Alemayehu et al., 2021; Ituma et al., 2019; Bulto et al., 2022; Odipe et al., 2019; Selepe & Mjoka, 2018; Teferi, 2022; Adane et al., 2018; Thandi & Campbell, 2011; Dagne et al., 2019; Abdalla et al., 2009; Nkhebenyane & Lues, 2020; Teffo & Tabit, 2020; Bigson et al., 2020; Zeleke et al., 2022; Tessema et al., 2014; Tamiru et al., 2022; Mariam et al., 2022; Yenealem et al., 2020; Matumba et al., 2016; Okojie et al., 2005; Isara et al., 2010; Omemu & Aderoju, 2008). All included studies were cross-sectional studies. Of these, 24 were community-based cross-sectional studies, while the remaining 18 studies were institutionally conducted. Of these cross-sectional studies, 32 used probability sampling, seven studies were non-probability studies, and three studies used both methods. The earliest study was conducted in 2005 (Okojie et al., 2005), and the most recent five articles (Fanta et al., 2023; Werkneh et al., 2023; Negassa et al., 2023; Engdaw et al., 2023; Alemu et al., 2023) were published in 2023. Eighteen studies conducted in Ethiopia (Fanta et al., 2023; Abegaz, 2022; Werkneh et al., 2023; Negassa et al., 2023; Engdaw et al., 2023; Teferi et al., 2021; Azanaw et al., 2019; Alemu et al., 2023; Tegegne & Phyo, 2017; Alemayehu et al., 2021; Bulto et al., 2022; Teferi, 2022; Adane et al., 2018; Dagne et al., 2019; Zeleke et al., 2022; Tessema et al., 2014; Tamiru et al., 2022; Yenealem et al., 2020), six studies in Nigeria (Oladoyinbo et al., 2015; Ituma et al., 2019; Odipe et al., 2019; Okojie et al., 2005; Isara et al., 2010; Omemu & Aderoju, 2008), six studies in South Africa (Makhunga et al., 2023; Marutha & Chelule, 2020; Mbombo-Dweba et al., 2022; Selepe & Mjoka, 2018; Nkhebenyane & Lues, 2020; Teffo & Tabit, 2020), three studies in Tanzania (Jumanne & Sophia, 2014; Nonga et al., 2014; Mariam et al., 2022), three studies in Ghana (Tuglo et al., 2021; Akabanda et al., 2017; Bigson et al., 2020), two studies in Malawi (Thandi & Campbell, 2011; Matumba et al., 2016), one study in Kenya (Mwove et al., 2020), one study in Sudan (Abdalla et al., 2009), one study in Cameroon (Blaise, 2014), and one study in Rwanda (Ndoli & Nicholas, 2019). The risk level of each study was assessed, and we found that all studies were rated as low risk of bias (Table 1).

**Table 1.** A descriptive summary of 42 studies in this systematic review and meta-analysis

Authors (Pub/Year)	Country	Study setting	Study design	Sample size	PFHP (%)	Sampling techniques	Study quality
Nguendo et al.(2014)	Cameroon	CB	CSS	837	45	Probability	Low risk
Johnson et al. (2020)	Kenya	IB	CSS	345	78.3	Probability	Low risk
Fresenbet et al. (2023)	Ethiopia	IB	CSS	284	42.6	Probability	Low risk
Silamlak et al. (2022)	Ethiopia	IB	CSS	291	48.8	Probability	Low risk
Oladoyinbo et al. (2015)	Nigeria	CB	CSS	473	43.8	Probability	Low risk
Adhena et al. (2023)	Ethiopia	CB	CSS	185	58.9	Probability	Low risk
Belay et al. (2023)	Ethiopia	CB	CSS	390	31.5	Probability	Low risk
Sophia et al. (2014)	Tanzania	IB	CSS	206	49.5	Probability	Low risk
Garedew et al. (2023)	Ethiopia	CB	CSS	417	37.6	Probability	Low risk
Lawrence et al. (2021)	Ghana	CB	CSS	407	62.9	Non-probability	Low risk
Earl et al. (2022)	South Africa	CB	CSS	252	80.4	Non-probability	Low risk
Samuel Chane et al. (2021)	Ethiopia	CB	CSS	422	50.5	Probability	Low risk
Khomotso et al. (2020)	South Africa	IB	CSS	312	66.2	Probability	Low risk
James et al. (2022)	South Africa	CB	CSS	40	72.5	Non-probability	Low risk
Jember et al. (2019)	Ethiopia	IB	CSS	384	49	Probability	Low risk
Ndoli et al. (2019)	Rwanda	IB	CSS	218	31.1	Probability	Low risk
Mekuriaw et al. (2023)	Ethiopia	CB	CSS	422	47.6	Probability	Low risk
Hezron et al. (2014)	Tanzania	CB	CSS	90	22.1	Non-probability	Low risk
Tegegne et al. (2017)	Ethiopia	IB	CSS	91	64	Probability	Low risk
Fortune et al. (2017)	Ghana	IB	CSS	235	83.8	Probability	Low risk
Tadege et al. (2021)	Ethiopia	IB	CSS	408	54	Probability	Low risk
Ituma et al. (2019)	Ethiopia	IB	CSS	68	70.6	Probability	Low risk
Tadesse et al. (2022)	Nigeria	CB	CSS	170	27.6	Probability	Low risk
Odipe et al. (2019)	Nigeria	CB	CSS	36	55.6	Non-probability	Low risk
Selepe et al. (2018)	South Africa	IB	CSS	19	36.3	Probability	Low risk
Samuel et al. (2022)	Ethiopia	CB	CSS	384	51.3	Probability	Low risk
Metadel et al. (2018)	Ethiopia	CB	CSS	116	53	Probability	Low risk
Penelope et al. (2011)	Malawi	CB	CSS	150	8.7	Probability	Low risk
Henok et al. (2019)	Ethiopia	CB	CSS	423	49.6	Probability	Low risk
Abdalla et al. (2009)	Sudan	CB	CSS	50	41.8	Non-probability	Low risk
Jane Sebolelo et al. (2020)	South Africa	IB	CSS	100	78.3	Probability	Low risk
Lesiba et al. (2020)	South Africa	IB	CSS	210	60	Non-probability	Low risk
Kate et al. (2020)	Ghana	IB	CSS	720	66.7	Probability and non-probability	Low risk
Agerie et al. (2022)	Ethiopia	CB	CSS	423	44.9	Probability	Low risk
Tessema et al. (2014)	Ethiopia	CB	CSS	406	52.5	Probability	Low risk
Sanbato et al. (2022)	Ethiopia	CB	CSS	450	55.1	Probability	Low risk
Mariam et al. (2022)	Tanzania	IB	CSS	375	72.3	Probability and non-probability	Low risk
Dawit et al. (2020)	Ethiopia	CB	CSS	214	66.4	Probability	Low risk
Limbikani et al. (2015)	Malawi	CB	CSS	805	33	Probability and non-probability	Low risk
Okojie et al. (2005)	Nigeria	IB	CSS	102	14.7	Probability	Low risk
Isara et al. (2010)	Nigeria	CB	CSS	350	37.5	Probability	Low risk
Omemu et al. (2008)	Nigeria	CB	CSS	87	31	Probability	Low risk

*Note: CB = Community-Based, CSS = Cross-Sectional Study, IB = Institutional-Based, PFHP = Prevalence of Food Hygiene Practices.*

### Quality assessment

After screening the relevant studies, the selected studies were appraised for methodological validity using Joanna Briggs Institute (JBI) appraisal tool for prevalence studies (Moola et al., 2017). The tool had a total of eight questions (Q1–Q8), and those studies with positive answers of more than 50% of the tool (i.e. ‘Yes’ for 5 or more questions of the JBI tool) were included in this meta-analysis. The scoring was done by two authors (YAA and KAG), with the discrepancies resolved with discussion and consensus. When the disagreement between the two authors was not resolved with discussion, the third author (NAG) involved was a breaker. During the appraisal of each primary study, more emphasis was given to the appropriateness of the study objectives, study design, statistical analysis, any source of bias, and its management methods. Studies were considered low risk when they scored 50% and above on the quality assessment indicators, as reported in Table 2.

**Table 2.** Quality assessment of the included studies using the Joanna Briggs Institute (JBI) quality appraisal criteria

Quality appraisal for included studies in this systematic review and meta-analysis										
Authors (year)	Criteria								S	QA
	Clearly defined inclusion criteria	Describing the study setting	Valid and reliable exposure measurements	Objective and standard criteria for measurement	Identified confounders	Strategies to deal with confounders	Valid and reliable outcome measurement	Appropriate statistical analysis		
Nguendo et al. (2014)	N	Y	Y	Y	N	N	Y	Y	5	LR
Johnson et al. (2020)	Y	Y	Y	Y	N	Y	Y	Y	7	LR
Fresenbet et al. (2023)	Y	Y	Y	Y	N	Y	Y	Y	7	LR
Silamlak et al. (2022)	N	Y	Y	Y	N	Y	Y	Y	6	LR
Oladoyinbo et al. (2015)	Y	Y	Y	Y	N	Y	Y	Y	7	LR
Adhena et al. (2023)	Y	Y	Y	Y	N	Y	Y	Y	7	LR
Belay et al. (2023)	Y	Y	Y	Y	N	Y	Y	Y	7	LR
Sophia et al. (2014)	N	Y	Y	Y	N	N	Y	Y	5	LR
Garedew et al. (2023)	N	Y	Y	Y	N	Y	Y	Y	6	LR
Lawrence et al. (2021)	Y	Y	Y	Y	N	Y	Y	Y	7	LR
Earl et al. (2022)	Y	Y	Y	Y	N	Y	Y	Y	7	LR
Samuel Chane et al. (2021)	N	Y	Y	Y	N	N	Y	Y	5	LR
Khomotso et al. (2020)	N	Y	Y	Y	N	N	Y	Y	5	LR
James et al. (2022)	Y	Y	Y	Y	N	Y	Y	Y	7	LR
Jember et al. (2019)	Y	Y	Y	Y	N	Y	Y	Y	7	LR
Ndoli et al. (2019)	Y	Y	Y	Y	N	N	Y	Y	6	LR
Mekuriaw et al. (2023)	N	Y	Y	Y	N	N	Y	Y	5	LR
Hezron et al. (2014)	N	Y	Y	Y	N	Y	Y	Y	6	LR
Tegegne et al. (2017)	Y	Y	Y	Y	N	Y	Y	Y	7	LR
Fortune et al. (2017)	Y	Y	Y	Y	N	Y	Y	Y	7	LR
Tadege et al. (2021)	N	Y	Y	Y	N	N	Y	Y	5	LR
Ituma et al. (2019)	N	Y	Y	Y	N	Y	Y	Y	6	LR
Tadesse et al. (2022)	Y	Y	Y	Y	N	Y	Y	Y	7	LR
Odipe et al. (2019)	Y	Y	Y	Y	N	Y	Y	Y	7	LR
Selepe et al. (2018)	N	Y	Y	Y	N	N	Y	Y	5	LR
Samuel et al. (2022)	Y	Y	Y	Y	N	Y	Y	Y	7	LR
Metadel et al. (2018)	Y	Y	Y	Y	N	Y	Y	Y	7	LR
Penelope et al. (2011)	N	Y	Y	Y	N	Y	Y	Y	6	LR
Henok et al. (2019)	Y	Y	Y	Y	N	Y	Y	Y	7	LR
Abdalla et al. (2009)	Y	Y	Y	Y	N	Y	Y	Y	7	LR
Jane Sebolelo et al. (2020)	N	Y	Y	Y	N	Y	Y	Y	6	LR
Lesiba et al. (2020)	Y	Y	Y	Y	N	Y	Y	Y	7	LR
Kate et al. (2020)	N	Y	Y	Y	N	N	Y	Y	5	LR
Agerie et al. (2022)	Y	Y	Y	Y	N	Y	Y	Y	7	LR
Tessema et al. (2014)	N	Y	Y	Y	N	Y	Y	Y	6	LR
Sanbato et al. (2022)	Y	Y	Y	Y	N	Y	Y	Y	7	LR
Mariam et al. (2022)	Y	Y	Y	Y	N	Y	Y	Y	7	LR
Dawit et al. (2020)	N	Y	Y	Y	N	Y	Y	Y	6	LR
Limbikani et al. (2015)	Y	Y	Y	Y	N	Y	Y	Y	7	LR
Okojie et al. (2005)	N	Y	Y	Y	N	Y	Y	Y	6	LR
Isara et al. (2010)	N	Y	Y	Y	N	N	Y	Y	5	LR
Omemu et al. (2008)	Y	Y	Y	Y	N	Y	Y	Y	7	LR

*Note: Y = Yes, N = No, S = Scores, OQA = Overall Quality Assessment, LR = Low Risk.*

### Risk of bias assessment

The tool developed by Hoy et al. was used to assess the risk of bias for each included study (Hoy et al., 2012). The tool consists of 10 items that assess four areas of bias: internal validity and external validity. Items 1–4 evaluate selection bias, non-response bias, and external validity. Items 5–10 assess measure bias, analysis-related bias, and internal validity. The tool included (Q1) population representation, (Q2) sampling frame, (Q3) methods of participant selection, (Q4) non-response bias, (Q5) data collection directly from subjects, (Q6) acceptance of case definition, (Q7) reliability and validity of study instruments, (Q8) type of data collection, (Q9) length of prevalence period, and (Q10) adequacy of numerator and denominator. Studies were classified as ‘low risk’ if 8 and above of 10 questions received a ‘Yes’, ‘moderate risk’ if 6 to 7 of 10 questions received ‘Yes’ and ‘high risk’ if 5 or lower of 10 questions received a ‘Yes’. Therefore, all included studies had a low risk of bias (Table 3).

**Table 3.** Risk of bias assessment of the included studies

Risk of bias assessment for included studies in this systematic review and meta-analysis												
Authors (year)	Criteria										Scores	Overall risk of bias
	External validity				Internal validity							
	Q1	Q2	Q3	Q4	Q5	Q6	Q7	Q8	Q9	Q10		
Nguendo et al. (2014)	N	Y	Y	Y	Y	N	Y	Y	Y	Y	9	Low risk
Johnson M.et al. (2020)	N	Y	Y	Y	Y	Y	Y	Y	Y	Y	9	Low risk
Fresenbet et al. (2023)	N	Y	Y	Y	Y	Y	Y	Y	N	Y	8	Low risk
Silamlak et al. (2022)	N	Y	Y	Y	Y	Y	Y	Y	N	Y	8	Low risk
Oladoyinbo et al. (2015)	N	Y	Y	Y	Y	Y	Y	Y	Y	Y	9	Low risk
Adhena et al. (2023)	N	Y	Y	Y	Y	N	Y	Y	N	Y	7	Low risk
Belay et al. (2023)	N	Y	Y	Y	Y	N	Y	Y	Y	Y	8	Low risk
Sophia et al. (2014)	N	Y	Y	Y	Y	N	Y	Y	Y	Y	8	Low risk
Garedew et al.(2023)	N	Y	Y	Y	Y	N	Y	Y	Y	Y	8	Low risk
Lawrence et al. (2021)	N	Y	Y	Y	Y	Y	Y	Y	N	Y	8	Low risk
Earl et al. (2022)	N	Y	Y	Y	Y	Y	N	Y	N	Y	7	Low risk
Samuel Chane et al. (2021)	N	Y	Y	Y	Y	N	Y	Y	Y	Y	8	Low risk
Khomotso et al. (2020)	N	Y	Y	Y	Y	N	Y	Y	Y	Y	8	Low risk
James et al. (2022)	N	Y	Y	Y	Y	Y	Y	Y	Y	Y	9	Low risk
Jember et al. (2019)	N	Y	Y	Y	Y	Y	Y	Y	Y	Y	9	Low risk
Ndoli et al. (2019)	N	Y	Y	Y	Y	N	Y	Y	Y	Y	8	Low risk
Mekuriaw et al. (2023)	N	Y	Y	Y	Y	N	Y	Y	Y	Y	8	Low risk
Hezron et al. (2014)	N	Y	Y	Y	Y	Y	Y	Y	Y	Y	9	Low risk
Tegegne et al. (2017)	N	Y	Y	Y	Y	Y	Y	Y	Y	Y	9	Low risk
Fortune et al. (2017)	N	Y	Y	Y	Y	Y	Y	Y	Y	Y	9	Low risk
Tadege et al. (2021)	N	Y	Y	Y	Y	N	Y	Y	Y	Y	8	Low risk
Ituma et al. (2019)	N	Y	Y	Y	Y	Y	Y	Y	Y	Y	9	Low risk
Tadesse et al. (2022)	N	Y	Y	Y	Y	Y	Y	Y	N	Y	8	Low risk
Odipe et al. (2019)	N	Y	Y	Y	Y	Y	Y	Y	Y	Y	9	Low risk
Selepe et al. (2018)	N	Y	Y	Y	Y	N	Y	Y	Y	Y	8	Low risk
Samuel et al. (2022)	N	Y	Y	Y	Y	Y	Y	Y	Y	Y	9	Low risk
Metadel et al. (2018)	N	Y	Y	Y	Y	Y	Y	Y	Y	Y	9	Low risk
Penelope et al. (2011)	N	Y	Y	Y	Y	Y	Y	Y	Y	Y	9	Low risk
Henok et al. (2019)	N	Y	Y	Y	Y	Y	Y	Y	Y	Y	9	Low risk
Abdalla et al. (2009)	N	Y	Y	Y	Y	Y	Y	Y	Y	Y	9	Low risk
Jane Sebolelo et al. (2020)	N	Y	Y	Y	Y	Y	Y	Y	N	Y	8	Low risk
Lesiba et al. (2020)	N	Y	Y	Y	Y	Y	Y	Y	N	Y	8	Low risk
Kate et al. (2020)	N	Y	Y	Y	Y	N	Y	Y	Y	Y	9	Low risk
Agerie et al. (2022)	N	Y	Y	Y	Y	N	Y	Y	N	Y	7	Low risk
Tessema et al. (2014)	N	Y	Y	Y	Y	Y	Y	Y	Y	Y	9	Low risk
Sanbato et al. (2022)	N	Y	Y	Y	Y	Y	Y	Y	Y	Y	9	Low risk
Mariam et al. (2022)	N	Y	Y	Y	Y	Y	Y	Y	N	Y	8	Low risk
Dawit et al. (2020)	N	Y	Y	Y	Y	Y	Y	Y	Y	Y	9	Low risk
Limbikani et al. (2015)	N	Y	Y	Y	Y	N	Y	Y	N	Y	7	Low risk
Okojie et al. (2005)	N	Y	Y	Y	Y	N	Y	Y	N	Y	7	Low risk
Isara et al. (2010)	N	Y	Y	Y	Y	N	Y	Y	N	Y	8	Low risk
Omemu et al. (2008)	N	Y	Y	Y	Y	Y	Y	Y	Y	Y	9	Low risk

*Note: Y = Yes, N = No.*

### Pooled prevalence of food hygiene practices in sub-Saharan Africa

**Figure 2.**
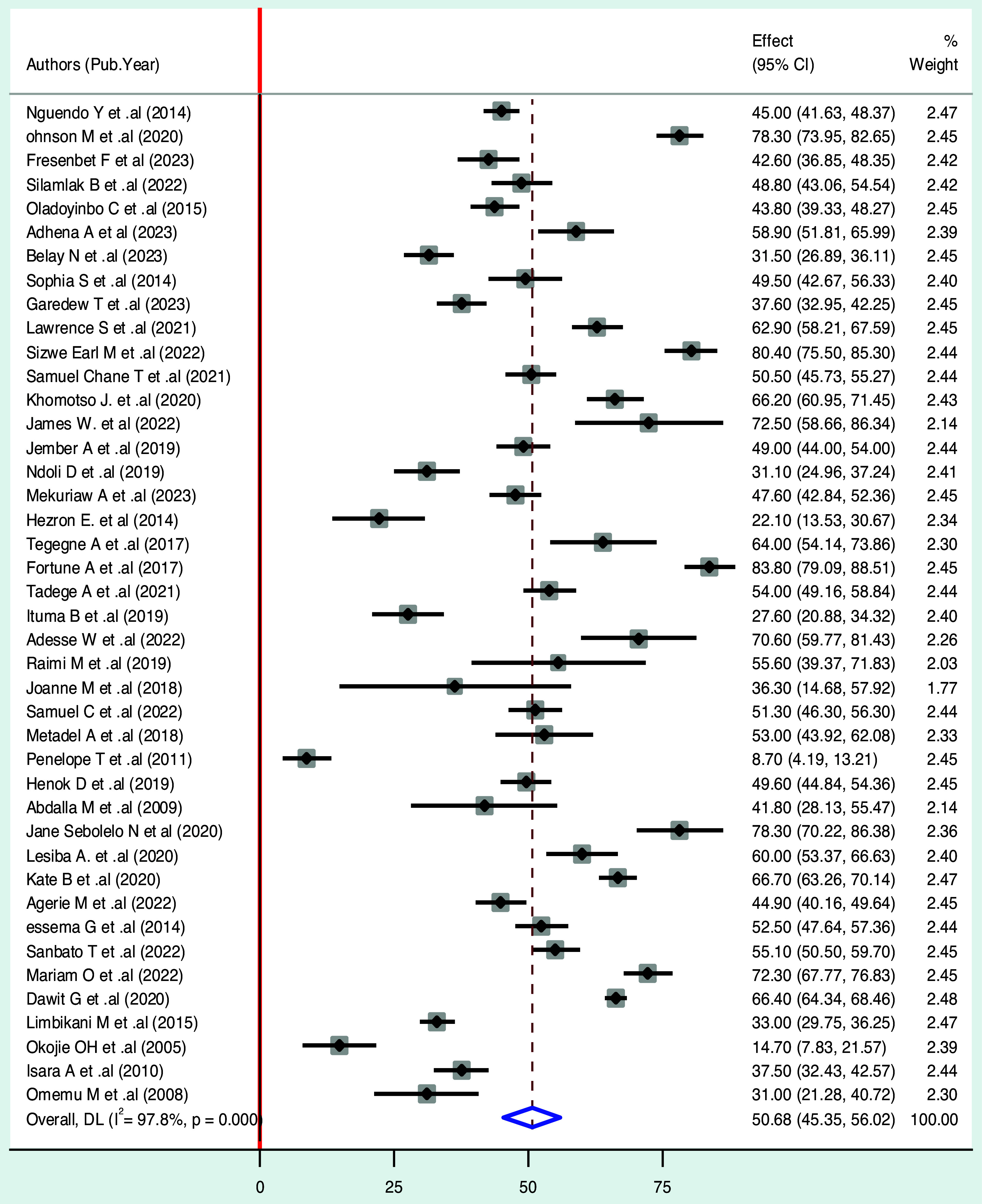
Forest plot displaying the pooled prevalence of food hygiene practice in sub-Saharan Africa.

A random effects model by DerSimonian and Laird was used to determine the overall pooled prevalence of food hygiene practices in sub-Saharan Africa. Accordingly, using a random effects model, the pooled prevalence of food hygiene practice among food handlers in sub-Saharan Africa was found to be 50.68% (95% CI: 45.35, 56.02) with a heterogeneity index (*I*^2^) of 97.8% (p < 0.001) (Fig. 2).

### Sub-group analysis for practices

Due to the significant heterogeneity observed, various factors were used to conduct sub-group analysis in this meta-analysis, including country, study setting, sample size, and sampling methods. Consequently, sub-group analysis revealed that the country of Kenya had the highest prevalence of hygienic food handling practices at 78.30% (95% CI: (73.95, 82.65)), followed by Ghana at 71.09% (95% CI: (59.41, 82.77)) and South Africa with 68.04% (95% CI: (58.99, 77.10)). In contrast, the lowest prevalence was observed in Malawi, where the prevalence of hygienic food handling practices was 20.90% (95% CI: (−2.911, 44.71)).

A sub-group analysis was performed based on the study sites. The prevalence of food hygiene practices was 55.72% (95% CI: (46.83, 64.61)) for institutional studies and 46.89% (95% CI: (40.19, 53.58)) for community-based studies. In addition, a sub-group analysis was conducted on studies using different sampling methods, including probability, nonprobability, and both. The prevalence of food hygiene practices in these studies was found to be 48.80% (95% CI: (42.84, 54.77)), 56.63% (95% CI: (42.13, 71.13)), and 57.30 % (95% CI: (32.28, 82.32)), respectively (Table 4).

**Table 4.** Sub-group analysis for the pooled prevalence of food hygiene practices in sub-Saharan Africa (n = 42)

Variables	Characteristics	Included studies	Sample size	Prevalence (95% CI)	Weights
Country	Cameroon	1	837	45.00 (41.63, 48.37)	2.47
	Kenya	1	345	78.30 (73.95, 82.65)	2.45
	Ethiopia	18	5,778	51.28 (46.12, 56.44)	43.50
	Nigeria	6	1,218	34.16 (24.40, 43.92)	14.01
	Tanzania	3	671	48.15 (20.21, 76.09)	7.19
	Ghana	3	1,362	71.09 (59.41, 2.77)	7.36
	South Africa	6	933	68.04 (58.99, 77.10)	13.54
	Rwanda	1	218	31.10 (24.95, 37.24)	2.41
	Malawi	2	955	20.90 (−2.911, 44.71)	4.92
	Sudan	1	50	41.80 (28.12, 55.47)	2.14
Study setting	Community-based	24	7,194	46.89 (40.19, 53.58)	57.25
	Institutional based	18	5,173	55.72 (46.83, 64.61)	42.75
Sampling techniques	Probability	32	9,382	48.80 (42.84, 54.77)	76.67
	Non-probability	7	1,085	56.63 (42.13, 71.13)	15.94
	Probability and non-probability	3	1,900	57.30 (32.28, 82.32)	7.39

### Sensitivity analysis

In addition to conducting sub-group analyses, we performed a sensitivity analysis by excluding each study to investigate the origin of heterogeneity. This analysis showed that omitting one study had no statistically significant effect on the overall evaluation of the studies (Table 5).

**Table 5.** Sensitivity analysis for the prevalence of food hygiene practices in sub-Saharan Africa

Study omitted	Estimate	95% CI
Nguendo et al.(2014)	50.826	(45.305, 56.346)
Johnson et al.(2020)	49.989	(44.701, 55.278)
Fresenbet et al.(2023)	50.884	(45.448, 56.320)
Silamlak et al.(2022)	50.730	(45.279, 56.180)
Oladoyinbo et al.(2015)	50.856	(45.391, 56.320)
Adhena et al.(2023)	50.482	(45.052, 55.912)
Belay et al.(2023)	51.165	(45.804, 56.527)
Sophia et al.(2014)	50.712	(45.276, 56.148)
Garedew et al.(2023)	51.011	(45.590, 56.432)
Lawrence et al.(2021)	50.377	(44.925, 55.828)
Earl et al.(2022)	49.940	(44.652, 55.227)
Samuel Chane et al.(2021)	50.687	(45.212, 56.163)
Khomotso et al.(2020)	50.296	(44.870, 55.722)
James et al.(2022)	50.207	(44.816, 55.599)
Jember et al.(2019)	50.725	(45.258, 56.192)
Ndoli et al.(2019)	51.168	(45.790, 56.546)
Mekuriaw et al.(2023)	50.760	(45.289, 56.230)
Hezron et al.(2014)	51.369	(46.017, 56.722)
Tegegne et al.(2017)	50.370	(44.96, 55.779)
Fortune et al.(2017)	49.853	(44.630, 55.077)
Tadege et al.(2021)	50.600	(45.126, 56.074)
Ituma et al.(2019)	51.251	(45.890, 56.612)
Tadesse et al.(2022)	50.223	(44.826, 55.619)
Raimi et al.(2019)	50.582	(45.184, 55.979)
Joanne et al.(2018)	50.942	(45.558, 56.327)
Samuel et al.(2022)	50.667	(45.198, 56.137)
Metadel et al.(2018)	50.628	(45.208, 56.048)
Penelope et al.(2011)	51.749	(46.833, 56.665)
Henok et al.(2019)	50.710	(45.235, 56.184)
Abdalla et al.(2009)	50.878	(45.478, 56.278)
Jane Sebolelo et al.(2020)	50.017	(44.647, 55.387)
Lesiba et al.(2020)	50.454	(45.021, 55.887)
Kate et al.(2020)	50.278	(44.833, 55.723)
Agerie et al.(2022)	50.828	(45.365, 56.290)
Tessema et al.(2014)	50.637	(45.163, 56.111)
Sanbato et al.(2022)	50.572	(45.091, 56.053)
Mariam et al.(2022)	50.141	(44.765, 55.517)
Dawit et al.(2020)	50.283	(44.817, 55.749)
Limbikani et al.(2015)	51.132	(45.792, 56.473)
Okojie et al.(2005)	51.568	(46.302, 56.833)
Isara et al.(2010)	51.013	(45.595, 56.430)
Omemu et al.(2008)	51.147	(45.758, 56.537)
**Combined**	**50.683**	**(45.349, 56.018)**

### Meta-registration

In addition to conducting sub-group and sensitivity analyses, meta-regression was performed to detect sources of heterogeneity by country, sampling method, and study setting. The meta-regression results revealed no apparent source of heterogeneity by sample size, sampling technique, and year of publication (Table 6).

**Table 6.** Meta-regression analysis of factors affecting between-study heterogeneity

Heterogeneity sources	Coefficients	Standard error	P-value
Country	0.9802332	0.1566685	0.901
Sample size	1.000223	0.0006687	0.740
Year of publication	0.996507	0.032622	0.915
Sampling techniques (method)	0.9481159	0.5281416	0.924

### Publication bias

**Figure 3.**
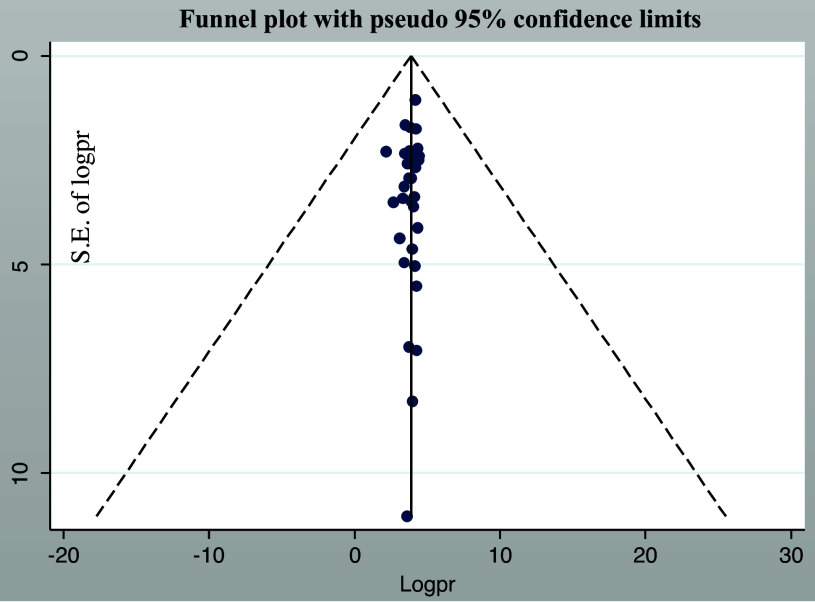
Forest plot displaying the asymmetrical distribution of the included studies.

The distribution of food hygiene practice was examined for asymmetry through a visual inspection of the forest plot presented as a funnel plot. Furthermore, Egger’s and Begg’s regression test results demonstrated the non-existence of publication bias (p = 0.31) and (P = 0.93), respectively (Fig. 3).

### Factors associated with food hygiene practice in sub-Saharan Africa

We performed a meta-analysis to identify associated factors for food hygiene practices using the random effects model. During the extraction process, we planned to show the association of each factor with the outcome variable. A total of 42 studies were included in the analysis of the factors associated with food hygiene practices. Therefore, we examined the pooled effect of four factors on the outcome variable such as food safety training, regular medical examinations, and levels of attitudinal factors.

Among 42 articles analyzed, seven studies indicated a significant association between hygienic food handling practices and food safety training (Abegaz, 2022; Werkneh et al., 2023; Tuglo et al., 2021; Azanaw et al., 2019; Alemu et al., 2023; Alemayehu et al., 2021; Adane et al., 2018). Findings revealed that individuals who underwent food safety training were 2.14 times more inclined to employ hygienic food handling practices compared to those who did not receive training 2.14 (OR: 2.14, 95% CI: (0.68, 6.76)).

Six articles indicated a significant association between attitudes and food hygiene practices (Abegaz, 2022; Werkneh et al., 2023; Tuglo et al., 2021; Alemu et al., 2023; Alemayehu et al., 2021; Yenealem et al., 2020). Findings revealed that individuals with a positive attitude were found to be 2.36 times more likely to adopt food hygiene practices than those with a negative attitude 2.36 (OR: 2.36, 95% CI: (1.36, 4.09)). These results highlight the importance of attitudes in promoting hygienic food handling practices.

In addition, four studies were analyzed to examine the association between handlers’ adherence to hygienic food handling practices and their regular medical examination, (Alemu et al., 2023; Teferi, 2022; Adane et al., 2018; Tamiru et al., 2022). Results revealed that individuals who did not receive routine medical checkups were discovered to have a 2.66 times higher likelihood of participating in unsanitary food handling behaviors compared to those who did undergo regular medical examination 2.66 (OR: 2.66, 95% CI: (1.52, 4.65)).

Moreover, in this study, seven articles were examined to determine the association between educational status and food hygiene practices. Findings revealed that educational status is not significantly associated with food hygiene practices at (P = 0.059). Six studies were used to estimate the association between knowledge and hygienic food handling practices among food handlers. Findings revealed that there is no significant relationship between knowledge and food hygiene hygienic practices at (P = 0.526). There was also a large heterogeneity (I^2^ = 96.0% and P = 0.001) among the included studies (Table 7).

**Table 7.** Factors associated with food handling practices among food handlers in sub-Saharan Africa

S.N.	Factors	Authors (pub. year) and *I*^*2*^ with P-value	Odd ratio (95% CI)
1	Educational status of food handlers	Belay et al.(2023)	3.42 (1.35, 8.64)
		Garedew et al.(2023)	1.50 (0.93, 2.40)
		Lawrence et al.(2021)	2.88 (1.81, 4.58)
		Samuel Chane et al.(2021)	3.42 (1.29, 9.05)
		Tadege et al.(2021)	1.23 (0.762,1.98)
		Samuel et al.(2022)	5.50 (1.05, 28.77)
		Agerie et al.(2022)	2.65 (1.21, 5.79)
		**Overall, DL (** ***I***^***2***^ ***=*** **50.6%, P =.059)**	2.23 (1.54, 3.22)
2	Food safety training for handlers	Silamlak et al.(2022)	0.09 (0.04, 0.18)
		Adhena et al.(2023)	0.49 (0.29, 0.82)
		Lawrence et al.(2021)	5.97 (3.50, 10.18)
		Jember et al.(2019)	4.01 (2.11, 7.61)
		Mekuriaw et al.(2023)	6.16 (2.97, 12.77)
		Tadege et al.(2021)	5.13 (3.46, 7.59)
		Metadel et al.(2018)	6.70 (1.80, 24.82)
		**Overall, DL (** ***I***^***2***^ ***=*** **96.0%, P =.000)**	2.14 (0.68, 6.76)
3	Level of knowledge of food handlers	Silamlak et al.(2022)	1.28 (0.49, 3.33)
		Adhena et al.(2023)	1.04 (0.43, 2.49)
		Sanbato et al.(2022)	2.32 (1.38, 3.89)
		Dawit et al.(2020)	2.04 (1.09, 3.81)
		Samuel Chane et al.(2021)	2.31 (1.53, 3.48)
		Tessema et al.(2014)	1.69 (1.04, 2.72)
		**Overall, DL (** ***I***^***2***^ ***=*** **0.0%, P = .526)**	1.96 (1.54, 2.44)
4	Attitude for food handlers	Dawit et al.(2020)	4.45 (2.09, 9.45)
		Adhena et al.(2023)	1.22 (1.00, 1.48)
		Silamlak et al.(2022)	1.19 (0.45, 3.12)
		Lawrence et al.(2021)	4.06 (1.63, 10.11)
		Mekuriaw et al.(2023)	3.55(1.14, 11.05)
		Tadege et al.(2021)	2.54 (1.51, 4.24)
		**Overall, DL (** ***I***^***2***^ ***=*** **78.6%, P = .000)**	2.36 (1.36, 4.09)
5	Regular medical checkup	Sanbato et al.(2022)	1.98 (1.14,3.43)
		Metadel et al.(2018)	5.20 (2.08, 12.98)
		Samuel et al.(2022)	3.87 (2.79, 5.36)
		Mekuriaw et al.(2023)	1.43 (0.82, 2.48)
		**Overall, DL (** ***I***^***2***^ ***=*** **76.3%, P = .005)**	2.66 (1.52, 4.65)

## Discussion

Food safety standards are the basis for controlling disease transmission from the food processor to the consumer (Uyttendaele, 2016). Food contamination and foodborne disease outbreaks are largely driven by food processors’ understanding and food hygiene practices, particularly in sub-Saharan Africa where food hygiene regulations are lax (Odeyemi, O. A. 2016). This systematic review aimed to determine the pooled prevalence of food hygiene practices and associated factors in sub-Saharan Africa.

In this study, the overall pooled prevalence of food hygiene practices among food handlers was found to be 50.68%. This result is almost consistent with earlier meta-analysis in Ethiopia (50.5%) (Zenbaba et al., 2022)). However, this finding is higher than studies conducted in Turkey (48.4%) (Mohlisi Mohd Asmawi et al., 2018). The disparity could be attributed to differences in procedure or variations in social cultural and personal hygiene practices. It might be also linked to inequitable sanitary conditions among food handlers, such as a lack of safe water and other sanitary facilities, which can contribute to poor adherence to food hygiene practices. Nevertheless, this finding is lower than the findings from Indonesia, (90%), Saudi Arabia (80.29%), Jordan (89.43%), and earlier meta-analysis study done in Ghana (55.8%) (Sharif & Al-Malki, 2010; Sharif et al., 2013; Lestantyo et al., 2017; Tuglo et al., 2023). The potential reason for this discovery may be attributed to the presence or absence of training opportunities, and in developing countries, several establishments operate without employing properly trained staff to handle food, and without implementing a system for conducting regular health assessments.

The prevalence of food hygiene handling practices in sub-Saharan Africa varies across countries, as considered by the sub-group analysis conducted in this study. These variations can be attributed to several factors such as socioeconomic conditions, environmental influences, and behavioral characteristics of food processors, and inequalities within countries due to differences in premises of food establishments. The included studies demonstrated significant heterogeneity due to differences in the training of study populations as an intervention and timing of outcome measures.

In addition, this study aimed to identify the factors associated with food hygiene practices among food handlers in sub-Saharan Africa. Accordingly, food safety training, regular medical examination, and a positive attitude were significantly associated with hygienic food handling practices. Food handlers who haven’t received food hygiene training are more likely to perform unsafe food handling than those who have received the training. This conclusion is supported by research conducted in Bangladesh (Rahman et al., 2016) and Malaysia (Mohlisi Mohd Asmawi et al., 2018). Training can improve the overall performance of food handlers in safe food handling. Therefore, food safety training appears to be a reliable indicator of food hygiene practices.

In this study, food handlers who exhibited positive attitudes were more likely to have food hygiene practices than those with negative attitudes. These results are consistent with previous studies conducted among food handlers in Brazil (Da Cunha et al., 2014) and Malaysia (Abdul-Mutalib et al., 2012). People who are more worried about the causes of foodborne diseases, and the consequences for their health make them engage in more protective behaviors (Mohlisi Mohd Asmawi et al., 2018). It is important to note that the attitude of food handlers plays a crucial role in translating food hygiene practices into observable measures, highlighting their influence on the level of handling practices.

Furthermore, regular medical examinations are associated with food hygiene practices, as evidenced by the fact that people who undergo medical examinations are more likely to demonstrate food handling practices compared to those who do not. This finding is consistent with previous research conducted in Bangkok, (Cuprasitrut et al., 2011). Healthcare workers who advised food handlers during the examination, enhancing their food handling practice and food handlers who are health-checked, have a better understanding of how to handle food safely. Therefore, workers undergo a medical examination before starting to work with food.

On the other hand, the combined findings of this meta-analysis shows no significant association between educational status and food hygiene practices. However, one earlier meta-analysis in Ethiopia examined, a significant association (Zenbaba et al., 2022). Other studies concluded, in support of the current study (Mohlisi Mohd Asmawi et al., 2018). Then, validating the concept of good hygienic food handling is primarily accomplished through effective food safety training for food handlers. Moreover, this finding showed that simply having knowledge about food hygiene does not necessarily translate into the implementation of safe food handling practices among individuals. In contrast, a meta-analysis performed in Ethiopia and Ghana found a significant association (Zenbaba et al., 2022; Tuglo et al., 2023). Various factors may contribute to this disconnect, including personal attitudes, cultural beliefs, and access to resources that facilitate proper food hygiene.

## Strengths and limitations of the study

This study was a first-of-its-kind systematic review and meta-analysis that estimated the pooled prevalence and associated factors of food hygiene practices in sub-Saharan Africa. To reduce the effects of selection bias, a systematic literature review was conducted focusing on clearly defined criteria. However, there are limitations to this study. We only searched papers published in English, and this study did not encompass qualitative research.

## Conclusion

In this study, only half of the food handlers in sub-Saharan Africa had good food hygiene practices. Lack of food safety training, a lack of regular medical checkups, and unfavorable attitudes toward food hygiene practices were all factors contributing to food hygiene practices. Thus, the authors recommended that food workers should have regular medical checkups and receive food safety training about food hygiene and safety procedures.

## Data Availability

The dataset and all the relevant files are found by the primary author and can be gained from the authors upon convincing request.
